# Evaluation of a Psychiatry of Later Life Consult Liaison Integration Initiative

**DOI:** 10.1192/j.eurpsy.2025.2439

**Published:** 2025-08-26

**Authors:** S. Crowley, M. Russell, N. McCarthy

**Affiliations:** 1 South Lee Mental Health Services, Cork University Hospital, Cork; 2 Clare Pscyhiatry Of Later Life, Gort Glas, Lifford Road, Ennis, Co. Clare, Ireland

## Abstract

**Introduction:**

Older people’s complex healthcare needs require the development of integrated mental and physical health services. The College of Psychiatrists of Ireland’s Faculty of Psychiatry of Old Age (POA) is in the process of drafting a position statement on the integration of POA with other services for older people such as the Integrated Care Program for Older People (ICPOP). ICPOP provides geriatric multi-disciplinary treatment in the community. In January 2024, the Clare Psychiatry of Later Life (POLL) team set up a novel consult liaison (CL) service with our local colleagues ICPOP colleagues.

**Objectives:**

The authors aim to describe a novel model of care for our newly developed ICPOP CL service. It may be of interest to other POLL teams who wish to expand their CL service to include their local ICPOP services or other community services. We also evaluated: referral patterns to the Clare POLL CL service, the number of referrals from the ICPOP CL service in its first six months, and key patient characteristics of ICPOP CL referrals compared to inpatient CL referrals.

**Methods:**

The model of care for our novel ICPOP CL clinic is described in detail. Patients were discussed at integrated multidisciplinary meetings. Cases brought by ICPOP for discussion with POLL could result in advice, a consult in a designated ICPOP CL clinic, or a referral for joint care. Joint teaching sessions were also arranged.

**Results:**

71.25 % (n=57, n=80) of referrals from Ennis General were for POLL CL. Conversely, CL referrals made up 21.43 % (n=81, n=378) of referrals to the Clare POLL service overall. ICPOP CL referrals made up 31.58% (n=18, n=57) of the POLL CL referrals over six months and made up 10% (n=18, n=180) of overall referrals to the Clare POLL service for the same period. Of these 18 ICPOP CL referrals, only four (22.22%) required joint care. Seven patients were referred by POLL to ICPOP for joint care over the same period. Patient characteristics were summarized by table 1.

**Image 1:**

**Figure:**
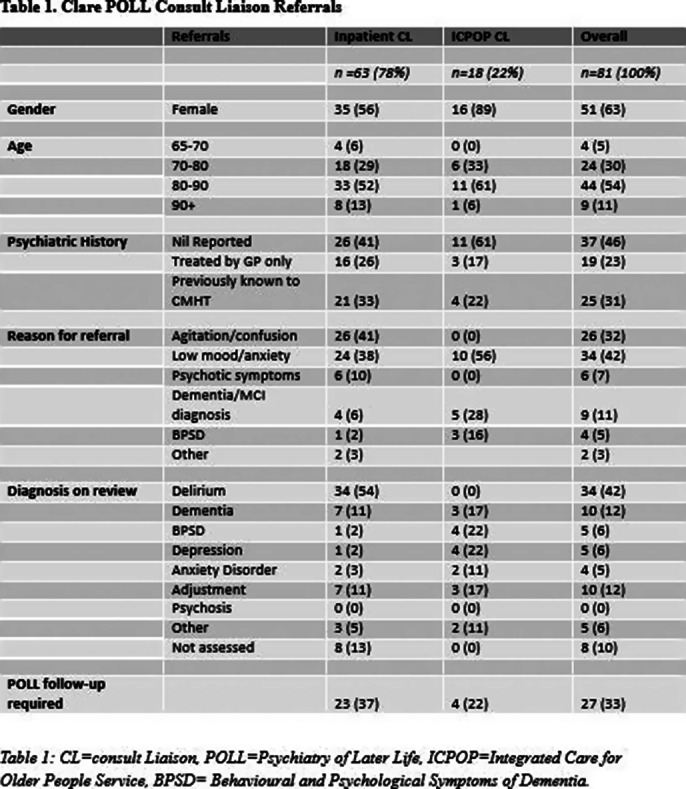

**Conclusions:**

The authors present a service integration initiative between a local POLL and ICPOP service, which they believe was mutually beneficial to both services and the patients they serve. The characteristics of patients referred to POLL from the new integration with ICPOP resemble other community referrals, as opposed to inpatient CL referrals.

**Disclosure of Interest:**

None Declared

